# Ammonia borane positively regulates cold tolerance in *Brassica napus* via hydrogen sulfide signaling

**DOI:** 10.1186/s12870-022-03973-3

**Published:** 2022-12-14

**Authors:** Pengfei Cheng, Liying Feng, Shuoyu Zhang, Longna Li, Rongzhan Guan, Weihua Long, Zhihui Xian, Jiefu Zhang, Wenbiao Shen

**Affiliations:** 1 College of Life Sciences, Laboratory Center of Life Sciences, Nanjing Agricultural University, Nanjing, 210095 Jiangsu China; 2 National Key Laboratory of Crop Genetics and Germplasm Enhancement, Jiangsu Collaborative Innovation Center for Modern Crop Production, Nanjing Agricultural University, Nanjing, 210095 Jiangsu China; 3 The Institute of Industrial CropsJiangsu Academy of Agricultural Sciences, Nanjing, 210014 Jiangsu China

**Keywords:** Ammonia borane, *Brassica napus*, Cold stress, Hydrogen sulfide, Oxidative stress

## Abstract

**Background:**

Cold stress adversely influences rapeseeds (*Brassica napus* L.) growth and yield during winter and spring seasons. Hydrogen (H_2_) is a potential gasotransmitter that is used to enhance tolerance against abiotic stress, including cold stress. However, convenience and stability are two crucial limiting factors upon the application of H_2_ in field agriculture. To explore the application of H_2_ in field, here we evaluated the role of ammonia borane (AB), a new candidate for a H_2_ donor produced by industrial chemical production, in plant cold tolerance.

**Results:**

The application with AB could obviously alleviate the inhibition of rapeseed seedling growth and reduce the oxidative damage caused by cold stress. The above physiological process was closely related to the increased antioxidant enzyme system and reestablished redox homeostasis. Importantly, cold stress-triggered endogenous H_2_S biosynthesis was further stimulated by AB addition. The removal or inhibition of H_2_S synthesis significantly abolished plant tolerance against cold stress elicited by AB. Further field experiments demonstrated that the phenotypic and physiological performances of rapeseed plants after challenged with cold stress in the winter and early spring seasons were significantly improved by administration with AB. Particularly, the most studied cold-stress response pathway, the *ICE1-CBF-COR* transcriptional cascade, was significantly up-regulated either.

**Conclusion:**

Overall, this study clearly observed the evidence that AB-increased tolerance against cold stress could be suitable for using in field agriculture by stimulation of H_2_S signaling.

**Supplementary Information:**

The online version contains supplementary material available at 10.1186/s12870-022-03973-3.

## Background

Plants are exposed to adverse environmental conditions frequently, facing a series of abiotic stresses, including salinity, osmotic, nutrient deficiency, metal stress, and extreme temperature [1]. Cold stress (low temperature) is a common environmental factor that inhibits plant growth and development, limits the geographical distribution of a species, and reduces crop yields [2, 3]. Many plant species from mid and high latitudes have evolved several distinct mechanisms to improve their cold tolerance during exposure to cold stress conditions [4]. Upon cold stress, several biochemical and physiological processes occur, ranging from the buildup of osmolytes and cryoprotectants for escaping the disturbance of reactive oxygen species (ROS) overproduction [5, 6]. Cold stress also influences much messenger molecules including phytohormones [7–10], metabolic enzymes [11], hydrogen peroxide (H_2_O_2_; [12]), nitric oxide (NO; [13]), and carbon monoxide (CO; [14]).

Hydrogen sulfide (H_2_S), the third gasotransmitters after NO and CO, was firstly studied in mammalian cells [15]. Although endogenous H_2_S production catalyzed by cysteine desulfhydrase (DES) activity and its release into atmosphere have been observed in higher plants [16, 17], the function and signaling of H_2_S in plants has been neglected for a long time. Further study showed that the H_2_S in plants is mainly enzymatically produced, including _L_-cysteine desulfhydrase (_L_-DES) and _D_-cysteine desulfhydrase (_D_-DES), sulfite reductase (SiR), cyanoalanine synthase (CAS), and *O*-acetyl-(thiol)-serinelyase (OAS-TL) [18]. Since the _L_-DES is primarily utilized for plant producing H_2_S [19], the enzyme _L_-DES has been also termed as DES [20]. In the last two decades, DES-dependent H_2_S has been progressively confirmed as an endogenous signaling molecule in plants [21–23], ranging from the regulation of plant development and the control of tolerance against various stresses [24]. Upon cold stress, the expressions and activities of DES were stimulated or increased in *Vitis vinifera* [25] and *Cucumis sativus* [26]. The underlying mechanisms partially include reconstructing redox homeostasis achieved by the interaction between H_2_S and other crucial molecules and pathways, including auxin [26], energy metabolism [27], antioxidant system [28], and mitogen activated protein kinase (MAPK; [29]).

Compared to H_2_S, molecular hydrogen (H_2_) was previously regarded as an important chemical material and most clean energy. In the last decade, combined with the progress in hydrogen biology in medicine [30], it is well-known that H_2_ might be one of the important gasotransmitters, controlling a diverse range of physiological events in a wide spectrum of biological systems [31]. In plants, the production of H_2_ is stimulated by several phytohormones (auxin and abscisic acid, etc.) and environmental stimuli to elicit some cellular processes. Ample evidence on the role of H_2_ in the plants has focused on the involvement of plant tolerance against abiotic stress, including salinity [32] and cold stress [33] as well as heavy metal exposure [34, 35]. In addition, H_2_S might be a crucial endogenous signal in H_2_ control of tolerance against osmotic stress [36] and prolonging the vase life of cut flowers [31].

Until now, two main methods of supplying H_2_ in biology are hydrogen rich liquid or its gas, and the applied H_2_ is mainly produced by electrolysis. Considering the future application in large scale agriculture, seeking a more convenient and safer H_2_ supply in crop-plantation, forestry, and animal husbandry, was a challenge for scientific community [37]. Ammonia borane (NH_3_ BH_3_; AB) is a potential alternative hydrogen donor in field of chemical industry [38] because of its high hydrogen capacity (19.6%) [39, 40]. In addition to H_2_, AB was hydrolyzed into trace amounts of ammonium ions and metaborate ions, both of which were beneficial for plant growth [41, 42]. Our previous results carried out in laboratory discovered AB control of rapeseed tolerance against osmotic stress, salinity, and cadmium exposure, and above achieved effects were similar to that with the conventional electrolytically produced HRW [35]. However, whether or how AB compound could be used to combat against cold stress is still elusive.

In this report, our laboratory and field experiments found AB control of cold tolerance in rapeseed via intensifying H_2_S signaling. Therefore, this work not only emphasized the important functions of H_2_S in hydrogen biology, but also provided a promising future of AB control of stress tolerance in field applications. We hope that the findings presented here will serve as an opportunity for the farmers and scientific community to push the hydrogen-based agriculture forward.

## Materials and methods

### Chemicals

Ammonia borane (AB) and sodium hydrosulfide (NaHS) as a solid hydrogen gas (H_2_) [35] and a hydrogen sulfid (H_2_S) donor [43], were purchased from Aladdin (Shanghai, China). Hypotaurine (HT), which was regarded as a H_2_S scavenger [44], and _DL_-propargylglycine (PAG), a chemical as an inhibitor of H_2_S synthesis [36], were purchased from Sigma (St Louis, USA). 7-azido-4-methylcoumarin (AzMC; [45]) used as a H_2_S fluorescent probe, was also purchased from Sigma (St Louis, USA).

Seeds of commercially available rapeseeds (*Brassica napus* L. Zhongshuang11) were sterilized with 5% (v:v) sodium hypochlorite solution for about 20 min, and washed with double distilled water for about 1 h. Afterwards, the uniform seeds were chosen and transferred to the plastic case and germinated in distilled water for 3 d in an incubator (temperature of 21 ± 1 °C, light intensity of 200 μmol^−1^·m^−2^·s^−1^ and 14 h photoperiod).

Three-day-old seedlings were kept at 21 °C (Con) or exposed to cold stress (Cold) condition (4 °C) [46] with or without 1 mg L^−1^ fresh AB [35], 1 mM NaHS [44], 500 μΜ HT [45], or 5 μΜ PAG [45] alone or the combination in incubators.

After treatments, the rapeseed seedlings were photographed, and experiment was carried with triplicates per experiment, and each replicate consisting of 50 plants, were used to detect phenotypes, or for other parameters.

### Determination of chlorophyll a and chlorophyll b contents

Chlorophyll in leaves (0.5 g) was isolated using 95% (v/v) ethanol for at least 48 h in darkness until the color of leaves fading, and contents of chlorophyll a and b were analyzed by absorbance detection at 665 nm (chlorophyll a) and 649 nm (chlorophyll b) [47]. Values are carried with three replicates for each experiment.

### Analyses of oxidative damage assay

Thiobarbituric acid reactive substances (TBARS) in root tissues were analyzed based on the methods as previously method [36]. The relative electrical conductivity (REC) in roots was analyzed by an electronic conductivity meter (DDS-12A; Kangyi Instrument, Shanghai, China), according to the previous method [33].

Hydrogen peroxide (H_2_O_2_) and superoxide anion (O_2_^.−^) in roots were spectrophotometrically analyzed, and histochemically stained with 3, 3’-diaminobenzidine (DAB) and nitroblue tetrazolium (NBT) followed the description from the previous studies [48]. Values in above experiment (except the staining) are from three independent replicates (0.5 g/treatment/repeat) for each experiment.

### Analyses of antioxidant enzymes analysis and oxidative damage assay

Superoxide dismutase (SOD), catalase (CAT), ascorbate peroxidase (APX), and peroxidase (POD) in the roots were determined according to the previous study [36]. And values are obtained from three independent replicates (0.5 g/treatment/repeat) for each experiment.

### Analyses of hydrogen sulfide (H_2_S) content and cysteine desulfhydrase (DES) activity

Endogenous H_2_S contents in root tissues were determined by using a spectrophotometric method or tracked in situ by LSM800 laser scanning confocal microscope (LSCM; Zeiss, Oberkochen, Germany) dependent on methylene blue from *N,N*-dimethyl-*p*-phenylenediamine or H_2_S-dependent fluorescent probe 7-azido-4-methylcoumarin, respectively (AzMC; [45]).

Cysteine desulfhydrase (DES) activity in root tissues was spectrophotometrically analyzed according to the formation of methylene blue [44].

Values in above experiment are from three independent replicates (0.5 g/treatment/repeat or 15 images/treatment/repeat) for each experiment.

### Real-time quantitative reverse transcription-PCR (qRT-PCR)

After the extraction of total RNA and the synthesis of cDNA from roots of seedlings and leaves collected in the field trials, a quantitative PCR (qRT-PCR) experiment was carried out. The primers’ sequences were shown in Supplementary Table S1. Relative expression levels of corresponding genes were normalized with two reference genes *Actin* and *GAPDH*, corresponding control samples. The results of relative genes expression levels were analyzed by the 2^−ΔΔ*C*T^ method [49].

### Field experiments

The *Brassica napus* L (*B*. *napus* L. cv. Zhongshuang11) was used in the field trails, which were planted in Nanjing, China by direct seeding in November of 2021 and the temperature of every day was recorded (Fig. S1). Thus, the seedlings were allowed to grow in the natural conditions and treated with or without AB once a month from December to February. There were two field groups (about 30 m^2^ for each treatment) which were irrigated with or without 1 mg L^−1^ AB.

### Statistical analysis

Values are presented as mean ± Standard Deviation (SD). Statistical analysis was performed using OriginPro 2021 (OriginLab Corporation, Northampton, Massachusetts, USA). Differences among treatments were analyzed by Turkey’s multiple range test, taking *P* < 0.05 as significant or *t* test (*P* < 0.01 or *P* < 0.001).

## Results

### Cold tolerance achieved by AB

**Fig. 1:**
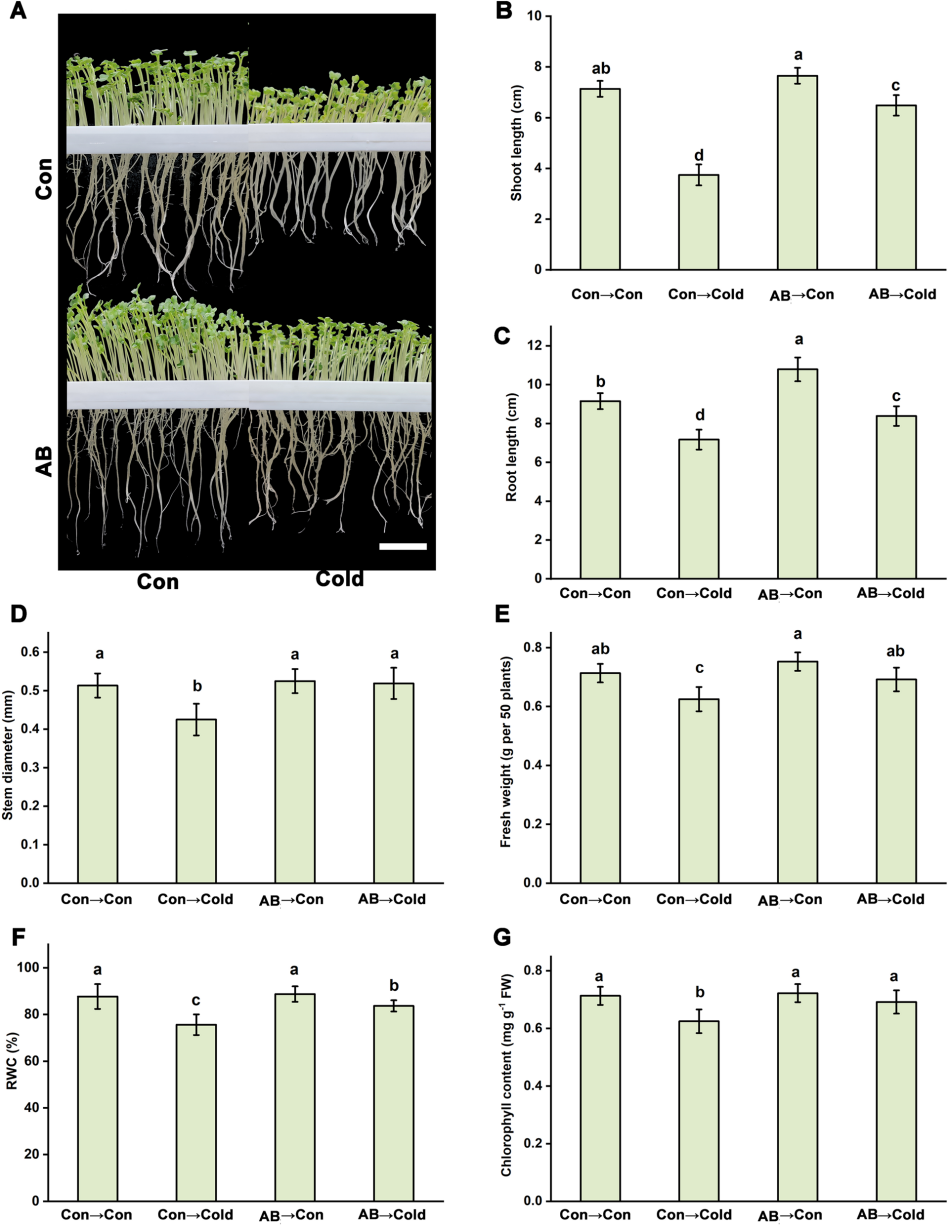
Cold tolerance achieved by AB. After germinating at room temperature (21 °C) for 3 days, rapeseed seedlings were kept at 21 °C or exposed to cold stress condition (4 °C) with or without 1 mg L.^−1^ AB treatment for another 3 days. Afterwards, corresponding photographs were taken (**A**). Meanwhile, the length of shoots (**B**) and roots (**C**), the diameter of stems (**D**), fresh weight of 50 plants (**E**), RWC (**F**), and chlorophyll content (**G**), were also determined. The error bars represent the SD. The different letters indicate significantly different values (*P* < 0.05 according to Turkey’s multiple range test)

Upon cold stress, rapeseed seedling growth was dramatically inhibited. However, the seedlings of AB-treated groups subjected to cold stress significantly alleviated the inhibitory impacts of cold damage (Fig. 1), relative to those in the normal temperature controls respectively, on shoots length (-10.8 ± 4.3% vs -35.6 ± 3.4% vs), roots length (-7.5 ± 4.6% vs -21.3 ± 3.8%), stem diameter (-2.8 ± 0.2% vs -19.8 ± 2.2%), fresh weight (-6.5 ± 0.3% vs -14.2 ± 2.8%), relative water content (RWC; -6.5 ± 0.5% vs -13.6 ± 1.6%), and chlorophyll contents (-3.2 ± 0.2% vs -14.8 ± 1.1%). Similar to the precious results [35], there were no significant different in the changes of seedlings growth (except root length) under the normal condition between regardless of AB addition.

### AB control of redox homeostasis in response to cold stress

**Fig. 2:**
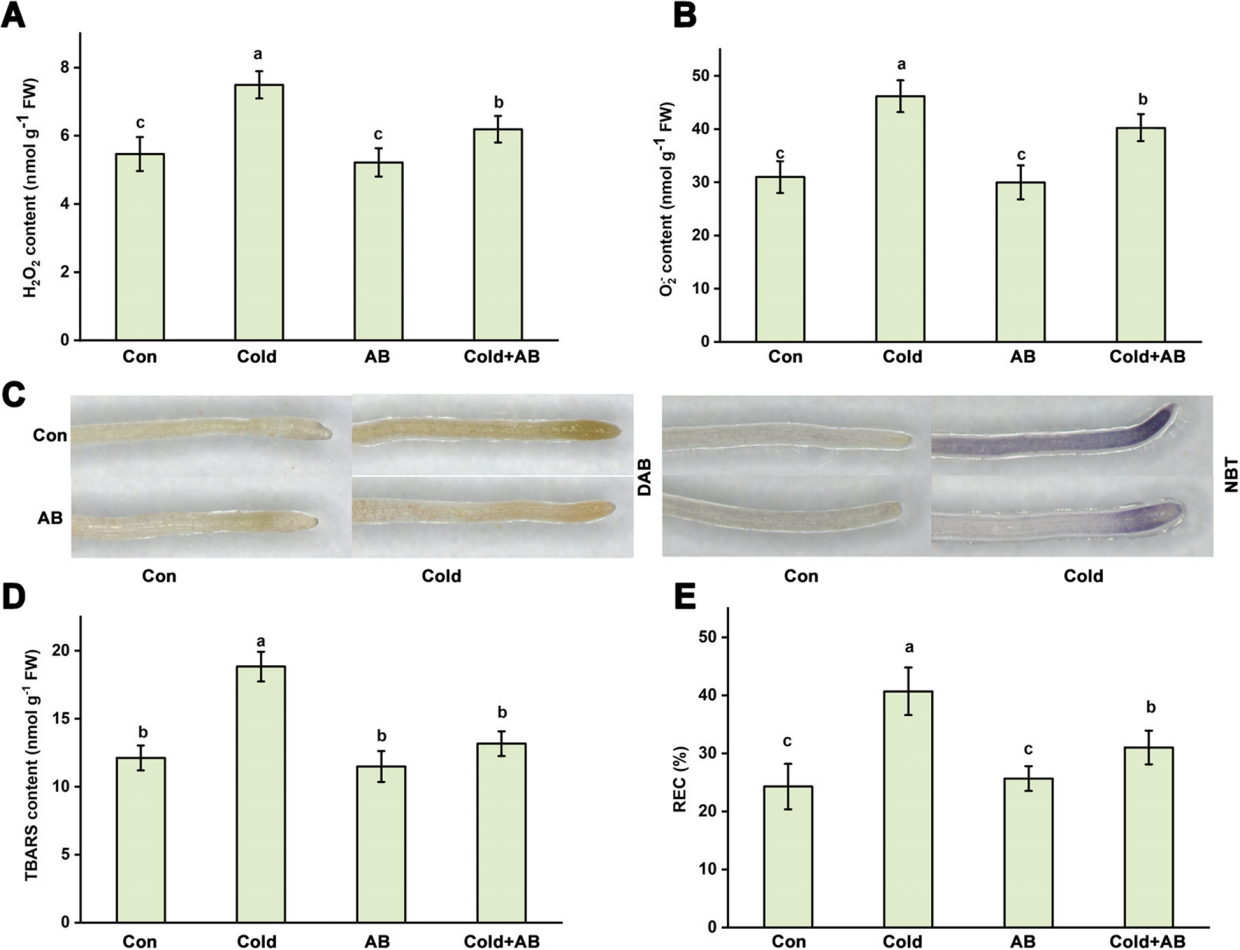
AB control of redox homeostasis in roots. After treatments for 3 days, the contents of H_2_O_2_ and O_2_.^.−^ were spectrophotometrically analyzed (**A**, **B**) and histochemically stained (**C**). Meanwhile, the contents of TBARS (**D**) and REC (**E**) were also measured. The error bars represent the SD. The different letters indicate significantly different values (*P* < 0.05 according to Turkey’s multiple range test)

It is well-known that the maintenance of redox homeostasis is crucial for plant survival in response to cold stress. We further investigated the role of AB in oxidative damage induced by cold stress. Compared with plants under the normal temperature, the changes in H_2_O_2_ and superoxide anion (O_2_^.−^) contents used to present oxidative damage, were sharply increased from about 5.46 ± 0.49 to 7.49 ± 0.40 and 30.98 ± 2.98 to 46.19 ± 2.99 mmol per g fresh weight (FW) respectively, in seedlings exposed to cold stress (Fig. 2A, B), reflecting the occurrence of oxidative damage caused by cold stress.

In contrast, AB addition could significantly decrease contents of H_2_O_2_ and O_2_^.−^ by about 24.1% and 20.5% compared with those in cold stress alone. Similar result was confirmed in the root tips stained with 3, 3’-diaminobenzidine (DAB) and nitroblue tetrazolium (NBT) (Fig. 2C), which were employed to visualize the distribution of H_2_O_2_ and O_2_^.−^, indicating that AB could regulate the reestablishment of redox balance upon cold stress. The altered levels of ROS suggested a potential change in lipid oxidation. As anticipated, changes in thiobarbituric acid reactive substances (TBARS) and relative electrical conductivity (REC) clearly indicated that lipid oxidation in root tissues was severely deteriorated upon cold stress conditions, which was significantly improved by the AB addition (Fig. 2D, E).

### Regulation of antioxidant defense by AB

**Fig. 3:**
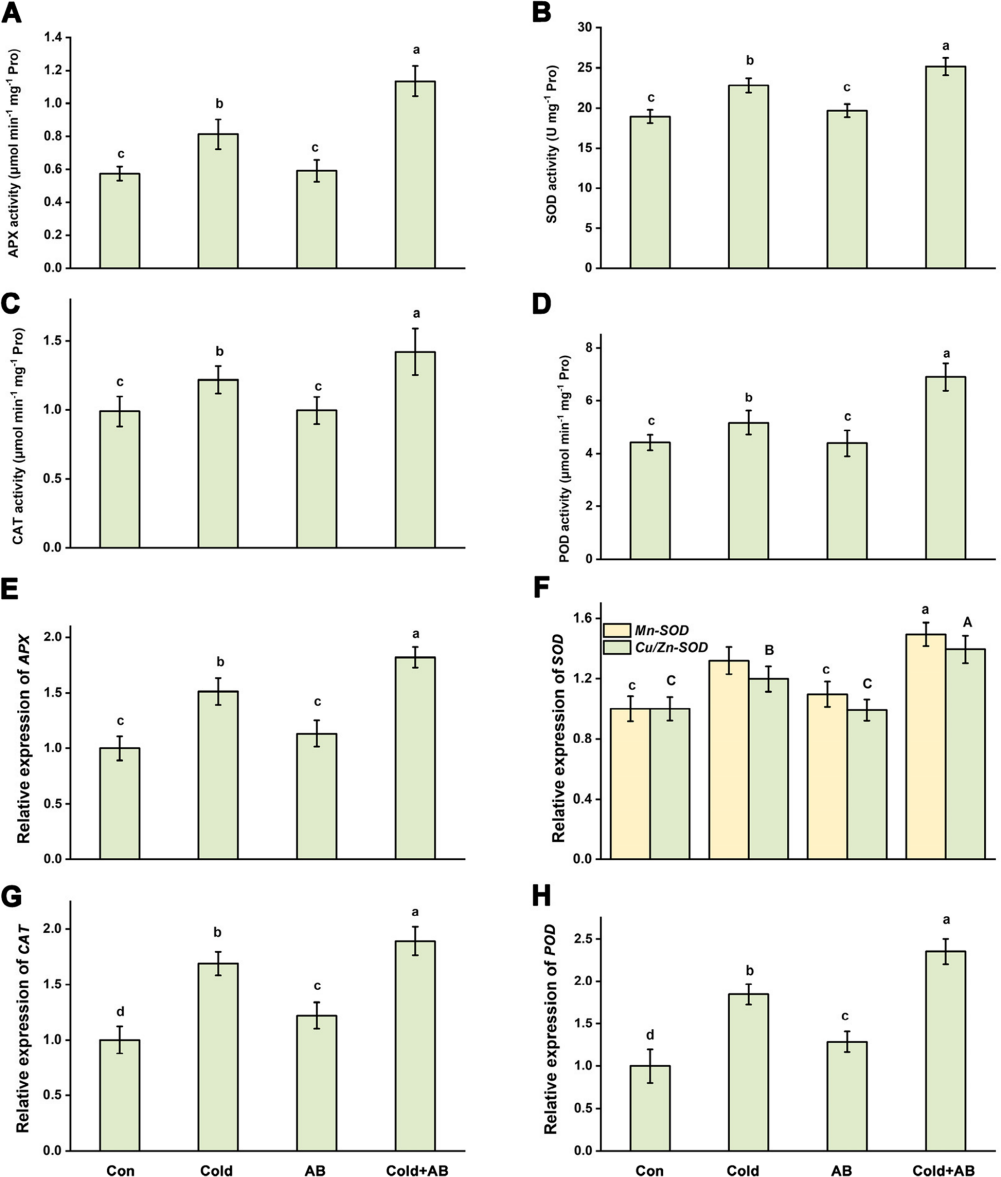
Regulation of antioxidant defense in roots. After treatments for 1 day, the activities of APX (**A**), SOD (**B**), CAT (**C**), and POD (**D**) were measured. Meanwhile, the transcriptional profiles of *APX* (**E**), *Mn-SOD* and, *Cu/Zn-SOD* (F), *CAT* (**G**), and *POD* (H) were analyzed. The error bars represent the SD. The different letters indicate significantly different values (*P* < 0.05 according to Turkey’s multiple range test)

To further elucidate related mechanism, the changes in antioxidant enzyme activities and corresponding transcripts were determined. As shown in Fig. 3A-D, cold stress significantly increased the activities of antioxidant system. Importantly, AB treatment could further increase above antioxidant enzyme activities in cold stressed plants, including APX (75.48 ± 10.59% vs 30.56 ± 8.75%), SOD (49.29 ± 9.32% vs 28.41 ± 6.75%), CAT (39.18 ± 4.49% vs 18.28 ± 3.04%), and POD (61.90 ± 11.18% vs 19.11 ± 5.25%), relative to the non-stressed controls, when compared with cold stress alone. Meanwhile, no significant change or weaker increment in above enzymatic activities was observed between AB alone and the normal growth condition. Importantly, the changes in transcriptional profiles of corresponding genes, including *APX*, *Mn-SOD*, *Cu/Zn-SOD*, *CAT*, and *POD*, displayed the similar tendencies (Fig. 3E-H), reflecting that AB regulated above antioxidant enzymes both at enzymatic and transcriptional levels when challenged with cold stress.

### Endogenous H_2_S production was intensified by AB

**Fig. 4:**
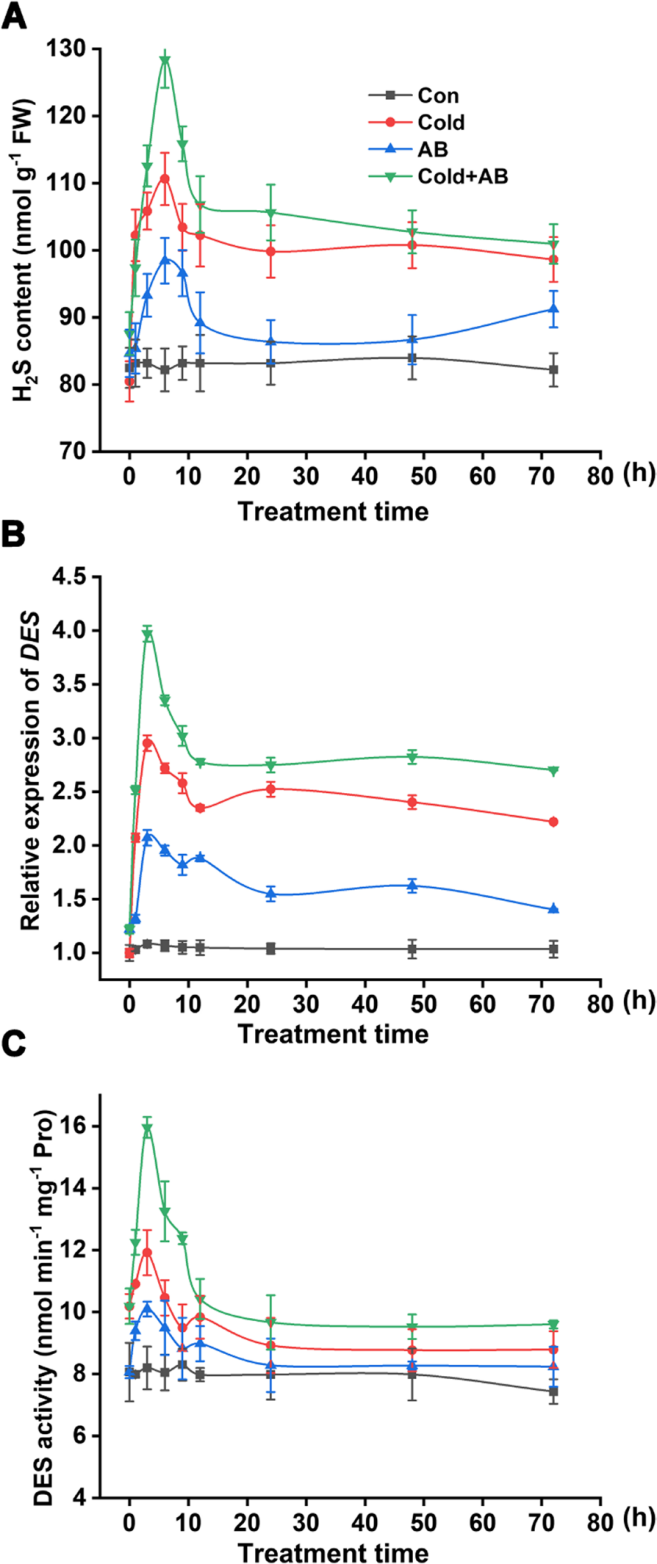
Cold-induced H_2_S production was positively regulated by AB. After treatments, changes in H_2_S content (**A**), transcriptional (**B**) and activity (**D**) levels of DES were analyzed. The error bars represent the SD

In order to assess the possibility of an inter-relationship between AB and H_2_S in plant tolerance against cold stress, the kinetics of H_2_S production in seedling roots after cold stress were analyzed. As expected, the basal level of H_2_S production as determined by spectrophotography in roots was stimulated upon cold stress during a 72-h period, showing a rapid and maximum increase of endogenous H_2_S after 6 h of treatment followed by a gradual decrease (Fig. 4A). We also clearly observed that cold stress-elicited H_2_S production was further strengthened by the AB addition, which was especially observed at a peaking time point. Interestingly, the changes in the transcripts and activities of DES (an important H_2_S synthetic enzyme) showed the similar tendencies, both of which peaked at 3 h of treatment, 3 h early than the H_2_S production (Fig. 4B, C). Above results provided a hypothesis that AB control of cold tolerance might be associated with H_2_S signaling.

### AB-induced cold tolerance achieved by the stimulation of H_2_S biosynthesis

**Fig. 5:**
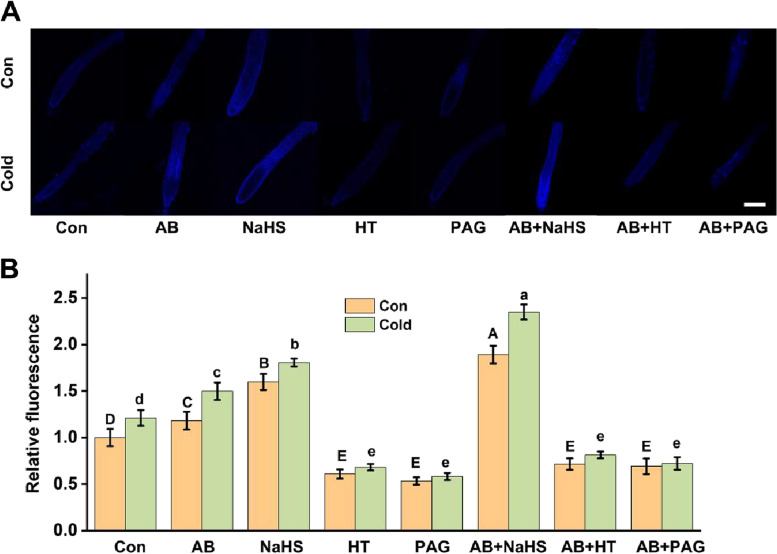
Altered endogenous H_2_S production by AB, PAG, and HT in response to cold stress. Three-day-old seedlings were kept at 21 °C (Con) or exposed to cold stress (Cold) condition (4 °C) with or without AB, NaHS, HT, and PAG alone and their combinations for 6 h. Afterwards, corresponding images of AzMC-dependent fluorescence in roots tips were provided to represent endogenous H_2_S contents (**A**), and the relative fluorescence was presented as values relative to Con (**B**). The error bars represent the SD. The different letters indicate significantly different values (*P* < 0.05 according to Turkey’s multiple range test)

In order to further verify the above hypothesis, the pharmacological manipulation of endogenous H_2_S levels was utilized to investigate a potential causal link between endogenous H_2_S and AB governing plant tolerance against cold stress. Here, NaHS (a well-known H_2_S donor), HT (a H_2_S scavenger), and PAG (an inhibitor of DES) were used individually or simultaneously together with AB in the presence or absence of cold stress. For endogenous H_2_S tracked in situ, a commercial specific fluorescent probe AzMC for H_2_S was applied together with confocal laser scanning microscopy. As expected, NaHS addition could increase AzMC-related florescent density in roots, and contrasting results were observed after the application with either HT or PAG (Fig. 5). These results clearly confirmed that the AzMC-dependent fluorescence is related to endogenous H_2_S level in rapeseed seedling roots; thus, this fluorescence was applied to report endogenous H_2_S level through the following study.

Similar to the results analyzed spectrophotometrically (Fig. 4A), cold stress-triggered AzMC-related florescent density was obviously stimulated in the presence of AB or NaHS individually or combination (especially) addition (Fig. 5). While, both HT and PAG inhibition of AB-induced florescent density in roots was also observed in the presence or absence of cold stress, compared to corresponding controls.

**Fig. 6:**
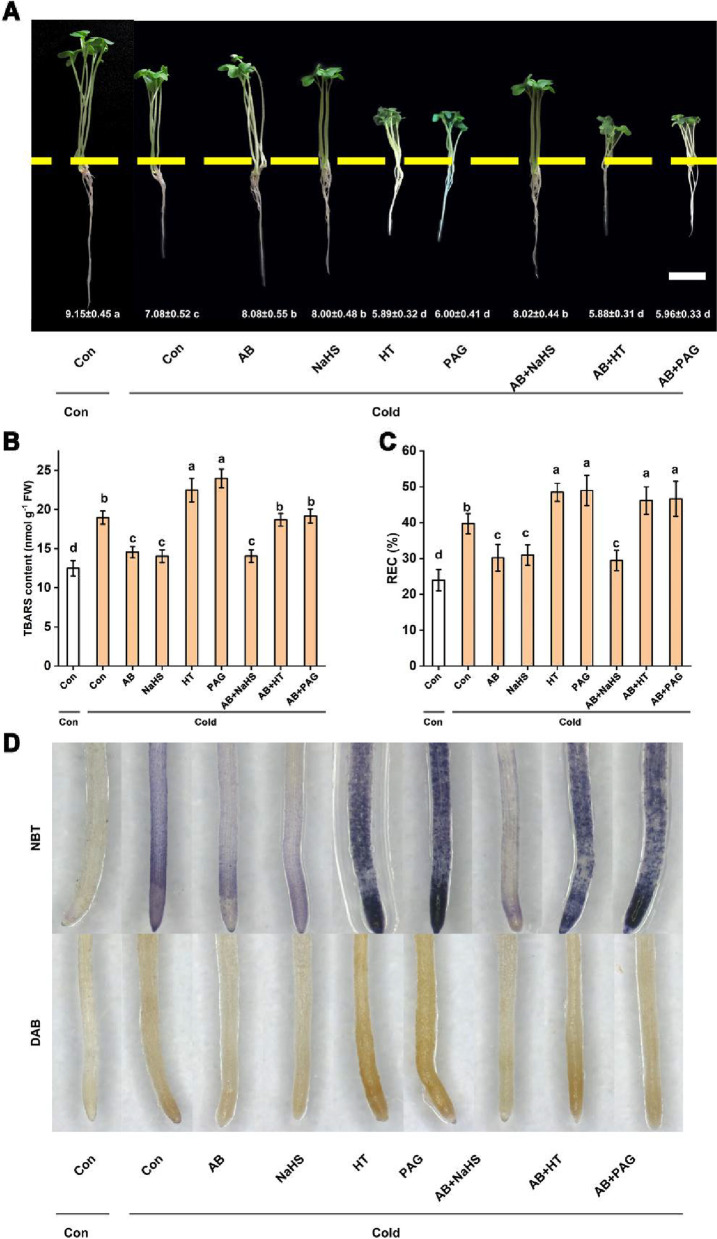
AB control of cold tolerance might be associated with the alteration of endogenous H_2_S production. After treatments for 3 d, the corresponding photos were taken, and changes in the root growth (**A**) were recorded. Meanwhile, the contents of TBARS (**B**) and REC (**C**) were also analyzed, and the distribution of O_2_.^.−^ and H_2_O_2_ (**D**) was histochemically determined. The error bars represent the SD. The different letters indicate significantly different values (*P* < 0.05 according to Turkey’s multiple range test)

Subsequent results showed that the alleviation of cold stress-induced growth inhibition and oxidative damage achieved by AB might be in a H_2_S-dependent fashion. For example, results shown in Fig. 6A revealed that compared to the stress alone plants, the addition with HT or PAG alone significantly strengthened the inhibition in root length, and the improving changes were observed when either NaHS or AB was added together with cold stress. Importantly, above effects achieved by NaHS or AB could be obviously abolished by the co-treatment with HT or PAG. We also noticed that in response to cold stress, unlike the additive role of the addition with NaHS and AB in the changes in endogenous H_2_S production (Fig. 5), no significant alteration in root length was discovered. Combined with changes in endogenous H_2_S, above results clearly indicated the important role of endogenous H_2_S homeostasis in the AB-conferred cold tolerance.

Further evaluation of the responses in oxidative damage revealed that both AB and NaHS alleviated the increases in TBARS (Fig. 6B) and relative electrical conductivity (REC; Fig. 6C). We also noticed that the AB- and NaHS-regulated reduction in H_2_O_2_ and O_2_^.−^ accumulation were visualized by histochemical staining in cold stress condition (Fig. 6D, E). The above positive effects achieved by AB and/or H_2_S were significantly impaired by the addition with HT or PAG. When applied alone, HT or PAG administration could intensify TBARS accumulation and REC, and increase H_2_O_2_ and O_2_^.−^ contents after cold stress. Contrasting responses were observed when NaHS was added after cold stress. Therefore, above results suggested that the AB-induced cold tolerance was achieved by the stimulation of H_2_S biosynthesis in rapeseed plants.

### Field experiments showed that AB positively regulates cold tolerance

**Fig. 7:**
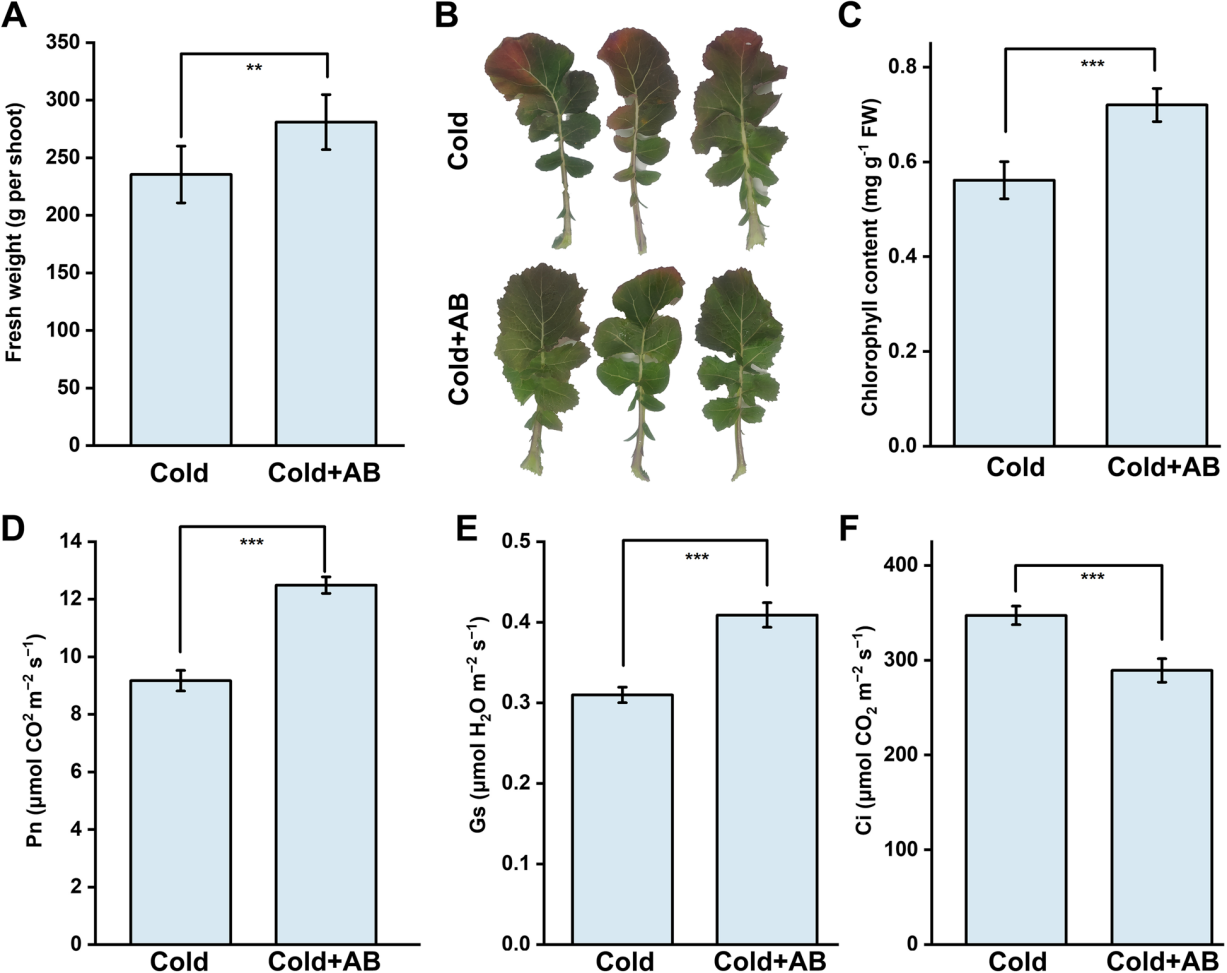
Field experiments showed that AB could be used to increase fresh weight and photosynthesis. During the winter season, seedlings were treated with or without AB for one month. At the early spring season (Feb, 16, 2022), the fresh weight of the shoot parts was measured, and the representative third leaves were chosen and imaged (**B**). Meanwhile, photosynthetic parameters, including total chlorophyll (**C**), Pn (**D**), Gs (**E**), and Ci (**F**) were measured. The error bars represent the SD. The ** or *** indicate significantly different values (*P* < 0.01, *P* < 0.001 according to t test)

**Fig. 8:**
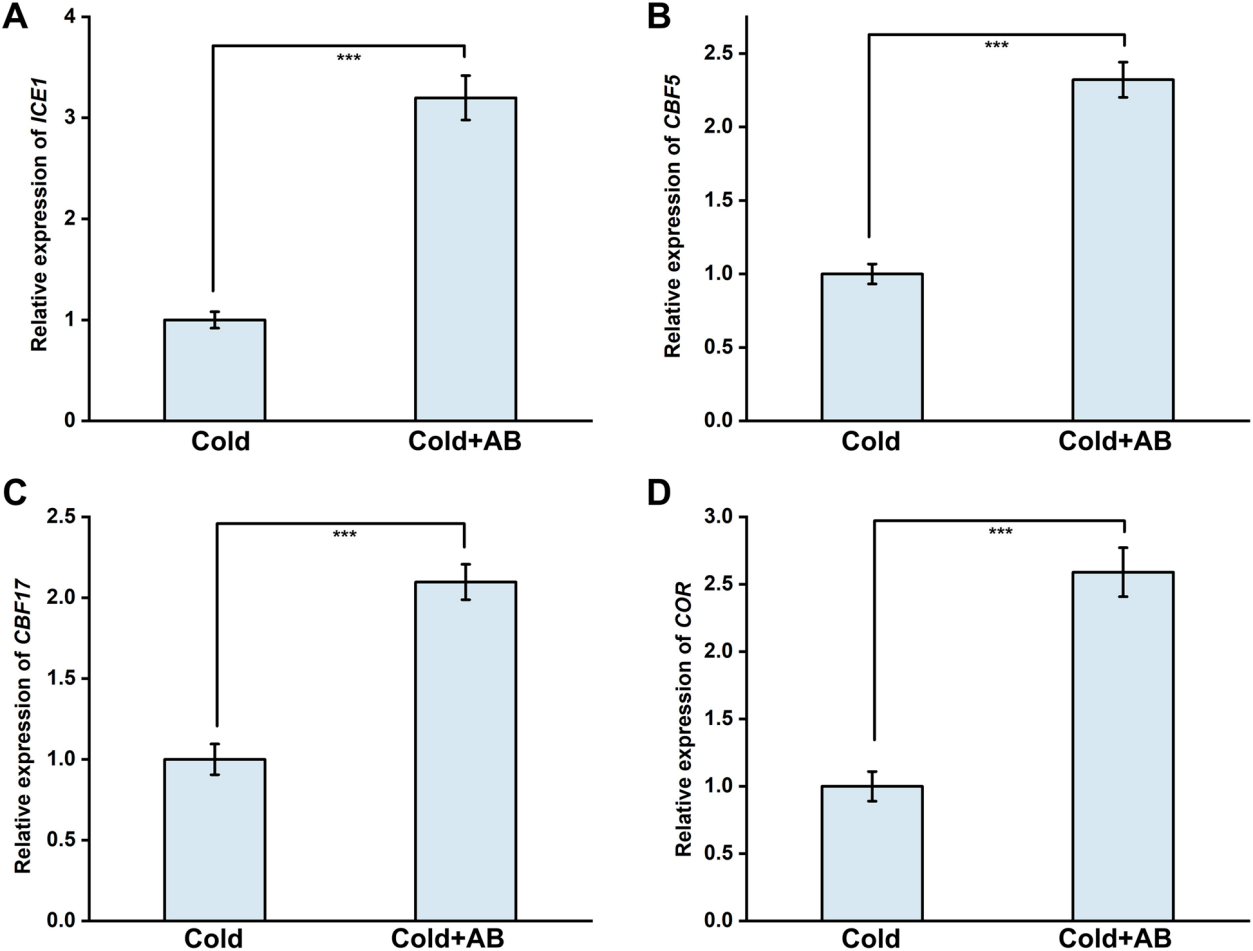
Cold response genes were positively regulated by AB in field. The third leaves were collected and used to extract RNA, and then the expression of some cold response genes, including *ICE1* (**A**), *CBF5* (**B**), *CBF17* (**C**), and *COR* (**D**) were analyzed by qPCR. The error bars represent the SD. The *** indicate significantly different values (*P* < 0.001 according to t test)

TO test the potential of AB used in agriculture, a field trial was conducted in Nanjing, Jiangsu Province, China from winter season (November) in 2021 to early spring (February, 2022). Similar with the results in laboratory experiments (Fig. 1A, B and G), the fresh weight of shoot parts and chlorophyll contents were negatively affected during cold temperature from winter and early spring seasons (Cold), but both of which were obviously enhanced by AB addition (Cold + AB; Fig. 7A-C). Meanwhile, net photosynthetic rate (Pn) and stomatal conductance (Gs) were also positively improved by applying AB, compared with AB-free plants (Fig. 7D-E). Meanwhile, the intercellular CO_2_ concentration (Ci) was decreased by the AB addition (Fig. 7F), obviously in the early spring. Similarly, further qRT-PCR results showed that the most studied cold-stress response pathway, the *ICE1-CBF-COR* transcriptional cascade (Chinnusamy et al., 2007), including the transcripts of *ICE1*, *CBF5*, *CBF17*, and *COR,* was significantly increased by the addition with AB (Fig. 8). All above results clearly showed that the AB administration could confer the adaptation of the field-grown rapeseeds against cold stress.

## Discussion

Normally, cold stress is an important environment factor that can substantially decrease the yield of crops to reduce their productivity, and limit their geographical distribution due to conditions often prevailing during the winter and early spring in the northern hemisphere [50–52]. When challenged with cold stress, since plants cannot run or hide, they develop some unique mechanisms to enhance their cold tolerance during cold acclimation [53, 54]. Similar to the responses of H_2_S [29, 43], we previously discovered that the increased production and subsequent action of H_2_ might be a key plant response to cold stress in alfalfa plants [33]. However, the biosynthetic pathway of H_2_ in cold stressed plants and mechanism underlying corresponding cold tolerance achieved by electrolytically produced HRW have not yet been fully elucidated. Most importantly, the short retention time of H_2_ in liquid solution and the expensive cost of the H_2_ supplied with conventional electrolytically produced HRW limit its large scale application in agriculture [55, 56].

In this study, several lines of evidence clearly suggested that exogenous application with AB, a solid and relatively idealized H_2_ donor in industry [38], could attenuate cold stress-induced rapeseeds growth and photosynthetic inhibition in both laboratory and field levels. The evidence includes: (i) simultaneous treatment with AB in both laboratory and field experiments obviously recovered the inhibited seedling growth and decreased photosynthesis caused by cold stress (evaluated by the changes in chlorophyll content and Pn, Gs, and Ci; Figs. 1, 7); (ii) cold stress-elicited oxidative damage was obviously abolished by AB via stimulating oxidative defense, which was evaluated by the increased activities and corresponding transcripts of representative antioxidant enzyme, including APX, SOD, CAT, and POD (Figs. 2, 3); and (iii) the *ICE1-CBF-COR* transcriptional cascade, a cold-stress response pathway [57], which has been confirmed to be coupled with H_2_S-dependent mitogen-activated protein kinase (MAPK) signaling transduction pathways upon cold stress [29], was stimulated by AB, and this result was obtained from a field experiment (Fig. 8). Combined with AB control of alfalfa tolerance against salinity, drought, and cadmium stress in laboratory experiments and the appropriate H_2_ releasing performance of AB chemical [35], we further deduced that AB might be used as a potential H_2_ donor in large scale agriculture.

How does AB mediate the induction of plant cold tolerance? Are other signals downstream of AB control of cold tolerance? Ample evidence has showed that H_2_S functions as a signal and bioregulator molecule in plant adaptive or responsive mechanism against abiotic stress, including cold stress [8, 26], salinity [45], and heavy metal exposure [58]. In our experiment conditions, cold stress-stimulated H_2_S synthesis in root tissues was further intensified by the AB addition (Fig. 4), and this result is a new finding. More specifically, we showed that above marked increase in endogenous H_2_S production achieved by AB resulted from enhanced DES activity due to the further up-regulation of *DES* gene expression. These results, together with that of Zhang et al. [36], highlight the novel function of DES in the mediation of H_2_S production elicited by H_2_. The requirement of DES in AB-intensified H_2_S synthesis was further confirmed by the findings that the addition of PAG, an inhibitor of DES enzyme [59], and HT, the scavenger of endogenous H_2_S [60], not only inhibited H_2_S production (Fig. 5), but also differentially abolished AB-recovered seedling growth inhibition caused by cold stress (Fig. 6A).

**Fig. 9:**
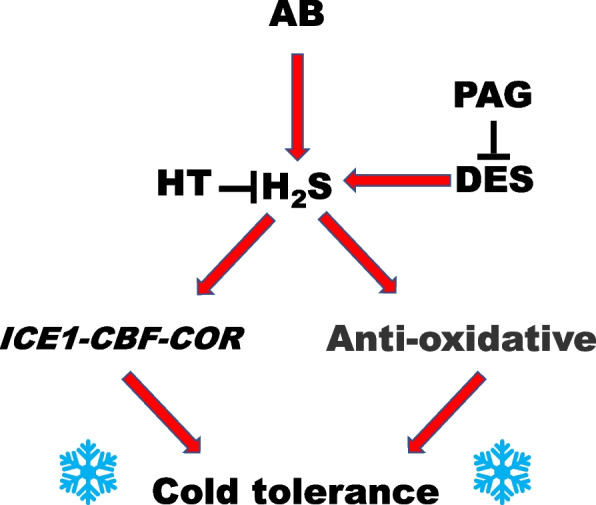
Schematic of the mechanism underlying ammonia borane (AB) positively regulating cold tolerance via hydrogen sulfide (H_2_S) signaling in *B*. napus. A rapid response of H_2_S production was observed after AB addition under cold stress. This enhanced H_2_S signal could enable plant to cope with cold stress via maintaining *ICE1-CBF-COR* pathway and redox homeostasis (especially)

Ample evidence confirmed that ROS not only act as signals [60, 62], but also have cytotoxic effects in both animals and plants, especially under the stressed conditions [63]. The increased activities of antioxidant enzymes, including SOD, CAT, APX, and GR achieved by H_2_S, were discovered in cucumber and pepper plants, which therefore regulated the ROS homeostasis and lipid peroxidation in response to salinity [18]. Notably, a series of constitutive proteins such as actin were included in the persulfidome [22]. Further study showed that the ROS level could be regulated by H_2_S via persulfidation of the NADPH oxidase [23]. Upon cold stress, an increased generation of ROS and induced lipid peroxidation were evident in various plant tissues [54, 61, 64]. In this study, we found that AB addition negatively altered the accumulation of ROS (H_2_O_2_ and O_2_^.−^) and TBARS as well as higher level of REC in cold-stressed conditions (Fig. 2). And most importantly, above responses achieved by AB were abolished by endogenous H_2_S deprivation by PAG or HT (Fig. 6B-D). Thus, combined with the physiological and biochemical parameters, it can be easily hypothesized that AB control of plant cold tolerance might be attributed to its ability to intensify H_2_S signal. These results were summarized in Fig. 9. In this model, AB-induced H_2_S homeostasis could participate in the process of the cold tolerance by maintaining *ICE1-CBF-COR* pathway and redox homeostasis (especially).

## Conclusion

In summary, this study clearly showed that AB can alleviate cold stress damage on rapeseed seedling growth inhibition (including root/shoot length, stem diameter, seedling weight, RWC, and chlorophyll content), observed in both the laboratory and partly in the field experiments. Specifically, the oxidative damage (expressed as the contents of H_2_O_2_ and O_2_^.−^) and member lipid peroxidation (represented by TBARS value and REC) triggered by cold stress were also significantly reduced by intensifying antioxidant defense (activities and transcriptional profiles of some antioxidase). Most importantly, we presented a novel signaling pathway where H_2_S acts downstream of AB governing cold stress in rapeseed plants.

On the other side, these findings expand our understanding on the roles of AB functionings in the regulation of plant physiology. Since AB is a powder which can be more easily transported and stored, as well as steadily used to release H_2_, both laboratory and field trials further confirmed the potential of the application of AB in a large-scale agricultural production.

## Supplementary Information

.

**Additional file 1: Supplementary Table 1.** The sequences of primers for qPCR. **Fig. S1.** During rape planting, the field daily average temperature and mean temperature in the past five years.

## Data Availability

All data generated or analyzed during this study are included in this published article.
