# Supplementary material for: Ammonia borane positively regulates cold tolerance in Brassica napus via hydrogen sulfide signaling
Source: BMC Plant Biol. 2022 Dec 14;22:585. doi: 10.1186/s12870-022-03973-3 (PMC9749201; doi:10.1186/s12870-022-03973-3)
Supplement: Supplementary file 1 — Additional file 1: Supplementary Table 1. The sequences of primers for qPCR. Fig. S1. During rape planting, the field daily average temperature and mean temperature in the past five years. [file 12870_2022_3973_MOESM1_ESM.docx]

**Supplementary Table 1** The sequences of primers for qPCR

| Gene name | Accession number | Forward primer | Reverse primer |
| --- | --- | --- | --- |
| *GAPDH* | [XM_009125769.3](https://www.ncbi.nlm.nih.gov/nuccore/1827811540) | TTGGTGACAACAGGTCAAGCA | AAACTTGTCGCTCAATGCAATC |
| *Actin* | [XM_009127096.3](https://www.ncbi.nlm.nih.gov/nuccore/1827681724) | TGTGCCAATCTACGAGGGTTT | TTTCCCGCTCTGCTGTTGT |
| *CAT* | [XM_013797971.2](https://www.ncbi.nlm.nih.gov/nuccore/1249689536) | GCCGAACCCGAAAACAAA | GTCATCAAACATCCAGCACCA |
| *Cu/Zn-SOD* | [XM_033281479.1](https://www.ncbi.nlm.nih.gov/nuccore/1827937873) | TCACTGACAGCCAGATTCCTC | CCTGCGTTTCCAGTAGACAA |
| *Mn-SOD* | [XM_009148545.3](https://www.ncbi.nlm.nih.gov/nuccore/1827836718) | TGGAAGCGTCTCCCTTGTTC | TTTCTCGGCCCATCTACGTG |
| *POD* | [XM_013859762.2](https://www.ncbi.nlm.nih.gov/nuccore/1249714582) | CTCCCACCGTATCCTCG | CGTGCTGCTCAAAGTCG |
| *APX* | [XM_009149789.3](https://www.ncbi.nlm.nih.gov/nuccore/1827887671) | CCCATTCGGAACAATGAGGT | ACAGCCACAACACCAGCAAG |
| *DES* | [XM_009106123.2](https://www.ncbi.nlm.nih.gov/nuccore/1827906827) | ATGGAAGAACGGTACATGATCAAGA | TCATTCAACCGGCAAATTCTCTGCT |
| *ICE1* | [XM_009153774.3](https://www.ncbi.nlm.nih.gov/nuccore/1827884185) | CCCGCCGGAAAACAACAACACAG | CGCCAGACGACGCCAAGACCTC |
| *CBF17* | [XM_013827830.2](https://www.ncbi.nlm.nih.gov/nuccore/1249658836) | AATGATCATGGCATGAACATGGC | ACTCCTCGTCCATGTAAAACCC |
| *CBF5* | [XM_009114740.3](https://www.ncbi.nlm.nih.gov/nuccore/1827924443) | GAGACAACATGCCCCAAGGA | TGTCCGAATTGTACGGACGG |
| *COR* | [XM_009105094.3](https://www.ncbi.nlm.nih.gov/nuccore/1827913582) | TCCGAATTCATGGCTATGTCTTTCTC | GGCGTCGACTCATGCCTTGTAGTGT |


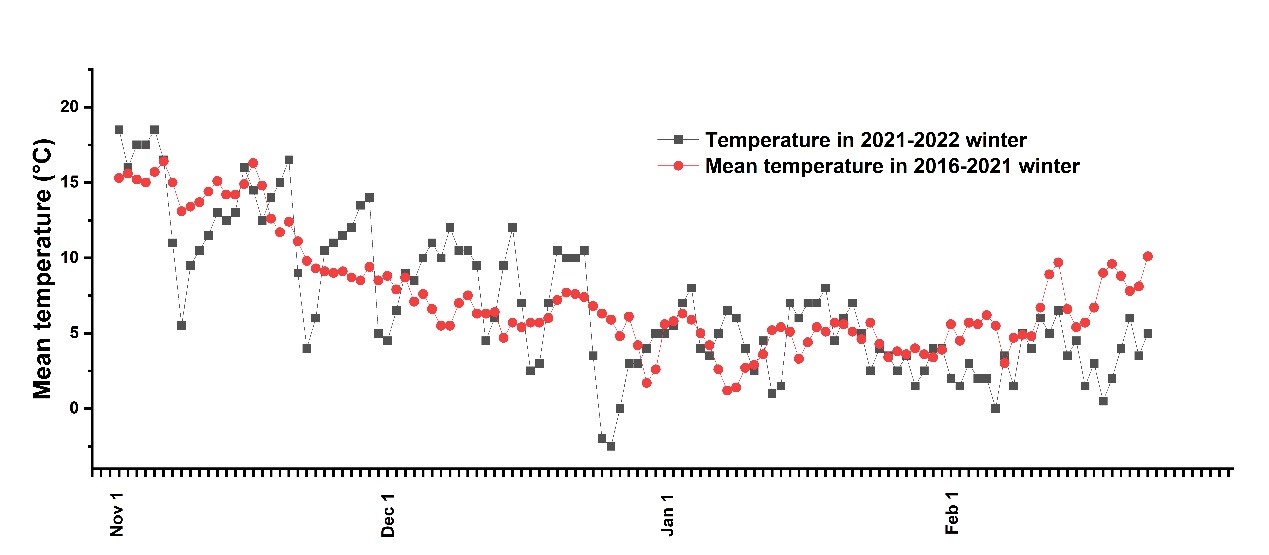
**Fig. S1**

Fig. S1. During rape planting, the field daily average temperature and mean temperature in the past five years.
